# d-Amino Acid Substitution of α-Conotoxin RgIA Identifies its Critical Residues and Improves the Enzymatic Stability

**DOI:** 10.3390/md17030142

**Published:** 2019-02-28

**Authors:** Jie Ren, Xiaopeng Zhu, Pan Xu, Rui Li, Ying Fu, Shuai Dong, Dongting Zhangsun, Yong Wu, Sulan Luo

**Affiliations:** Key Laboratory of Tropical Biological Resources, Ministry of Education, Key Lab for Marine Drugs of Haikou, Hainan University, Haikou 570228, China; hndx2303@163.com (J.R.); zhuxiaopeng@hainu.edu.cn (X.Z.); xpxgc@163.com (P.X.); flychcken@163.com (R.L.); fuying926@163.com (Y.F.); dongshuai_1024@163.com (S.D.); zhangsundt@163.com (D.Z.)

**Keywords:** d-amino acid scan, α-conotoxin RgIA, α9α10 nAChR, activity, stability

## Abstract

α-Conotoxin RgIA is a selective and potent competitive antagonist of rat α9α10 nicotinic acetylcholine receptors (nAChR), but it is much less potent towards human α9α10 nAChR. Furthermore, RgIA is susceptible to proteolytic degradation due to containing four arginine residues. These disadvantages greatly limit its use for clinical applications. The purpose of this research was to identify critical stereocenters of RgIA and discover more stable analogues, enhancing its bioavailability by using the d-amino acid scan method. The activity of each variant was investigated against rat and human α9α10 nAChRs, which were expressed in *Xenopus oocytes*. Experimental assays showed that 14 out of 15 analogues had a substantial reduction in potency towards rat α9α10 nAChR. Noticeably, analogue **13** retained full biological activity compared with RgIA. Meanwhile, two other analogues, **14** and **15**, of which l-Args were substituted with d-Args, exhibited a significantly increased potency towards human α9α10 nAChR, although these analogues showed decreased activities against rat α9α10 nAChR. Additionally, these three analogues exhibited a high resistance against enzymatic degradation in human serum and simulated intestinal fluid (SIF). Collectively, our findings suggest that a d-amino acid scan is a useful strategy for investigating how the side-chain chirality of amino acids affects the structure and function of peptides and may facilitate the development of more stable analogues to increase therapeutic potential.

## 1. Introduction

Venoms from the *Conus* species comprise a mixture of peptides called conotoxins (CTxs) that target voltage- and ligand-gated ion channels with unparalleled selectivity and potency [1,2,3]. These excellent properties provide potential for development as neuropharmacological probes or leads for drug development. In particular, ω-CTx MVIIA isolated from the venom of *Conus magus* targets a specific voltage-gated calcium channel subtype (Cav 2.2) and was approved by the United States Food and Drug Administration (FDA) and European Medicines Agency (EMA) as a novel type of analgesic, whereas some other CTxs have entered clinical or preclinical trials [4,5,6].

α-CTx RgIA is composed of 13 amino acids, belonging to the 3/4 subgroup, and was initially cloned from *Conus regius* [7]. This peptide has an extremely high affinity and selectivity for the rat α9α10 nAChR, which is an available receptor for investigating neuropathic pain treatment [8,9,10]. In various rat analgesic models, RgIA and its derivative RgIA4 have been proven to be effective antagonists, preventing neuropathic pain [11,12,13,14]. Due to its application potential, several studies have focused on the blocking molecular mechanism of RgIA, to guide the development of novel analogues [7,15,16,17]. However, the precise interaction sites of RgIA with α9α10 nAChR have not been fully characterized yet. The D-scan strategy has previously been applied to several peptides to reveal the critical amino acid involved in the peptide-receptor interaction [18,19,20,21]. Here, we applied this approach to synthesize a series of d-Amino Acid substitutions to assess the importance of the stereocenters in RgIA when binding to α9α10 nAChR.

Although RgIA has the potential for development as a novel analgesic in animal models, it also suffers from a poor stability and short biological half-life in vivo. Furthermore, RgIA has ~300-fold less potency on human versus rat α9α10 nAChR [22,23]. Additionally, RgIA is more affected by these problems compared to other conopeptides since it contains four arginine residues. Hence, two promising chemical approaches were applied to improve the stability of RgIA with a disulfide bond mimetic and backbone cyclization [24,25]. Research has also shown that the rational substitution of D-amino acids in some peptides can improve their stability against proteolytic attack [19,26,27,28]. However, this strategy has not been successfully applied to conotoxins up to now [29]. Inspired by these studies, we expected to create RgIA derivatives incorporating D-amino acids that would preserve original activity and be more stable than the wild-type peptide.

**Figure 1:**
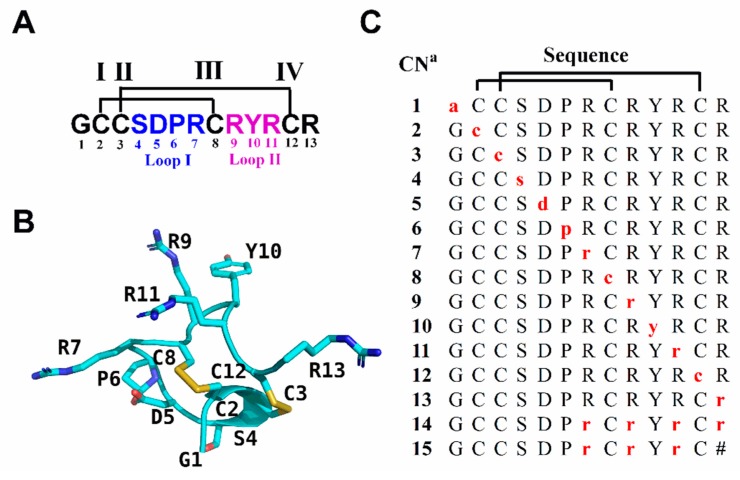
Sequence and structure of RgIA and its d-amino acid scan analogues. (**A**) The sequence of RgIA. Disulfide connectivity of CysI-CysIII and CysII-CysIV is labeled with black lines. Amino acids in loop I and loop II are marked in blue and purple, respectively. (**B**) NMR structure of RgIA (PDB ID 2JUT) [30]. (**C**) d-amino acid scan analogues of RgIA. The amino acids indicated by red lower case letters are d-amino acids. ^a^ Compound number. The # indicates a C-terminal amide.

In this study, we prepared a series of d-amino acid substitutions of RgIA and the glycine-1 at N-terminal was replaced by d-alanine due to its achirality (Figure 1). For peptides **14** and **15**, all l-Args were substituted by their d-amino acid counterparts. In particular, peptide **15** was designed because previous studies showed that the arginine-13 at C-terminal did not affect the binding activity of RgIA [25,30]. Then, these variants were tested for their functional activity against different kinds of nAChR subtypes. According to the results of electrophysiological assays, we picked several pivotal analogues to investigate their stability in human serum and simulated intestinal fluid (SIF). We found that this abolished the activity of most analogues, while several of them retained partial potency. Only one mutant, peptide **13**, kept its capacity of blocking α9α10 nAChR completely. Strikingly, the three variants **13**, **14**, and **15** were resistant to degradation in serum or SIF. Our research showed that the D-scan strategy can be used effectively to explore the important chiral centers in CTxs, and appropriate substitution with d-amino acids in CTxs is an efficient strategy to improve the stability and enhance their pharmaceutical applications.

## 2. Results

### 2.1. Synthesis and Oxidative Folding of RgIA Analogues

**Figure 2:**
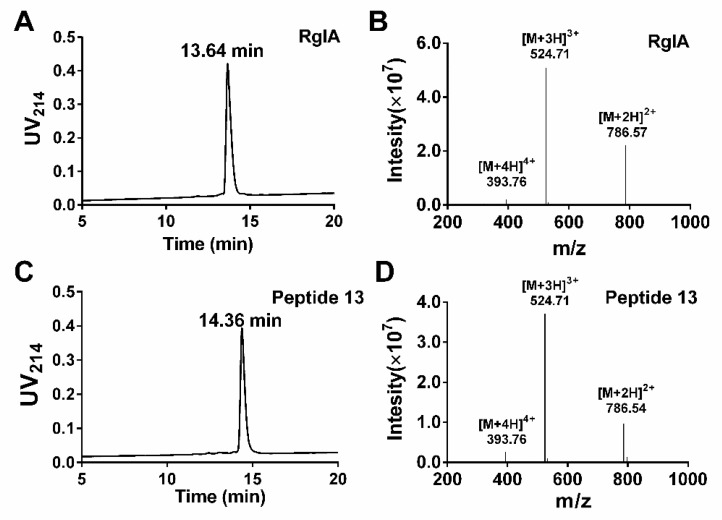
Analytical RP-HPLC and ESI-MS analysis of RgIA and **13**. The peptides were determined on a Vydac C18 column (4.6 × 250 mm, 5 μm) with a flow rate of 1 mL/min. The gradient of RP-HPLC was 10% buffer B ramping linearly to 40% over 20 min, where buffer A is 0.65% trifluoroacetic acid (TFA) in water, and buffer B is 0.5% TFA and 90% acetonitrile in water. UV detection was performed at 214 nm. (**A**) HPLC chromatogram of RgIA with a retention time of 13.64 min. (**B**)ESI-MS profile of RgIA with a mass of 1571.13 Da. (**C**) HPLC chromatogram of peptide **13** with a retention time of 14.36 min. (**D**) ESI-MS profile for peptide **13** with a mass of 1571.13 Da.

We used the d-amino acid scanning mutagenesis method to identify critical residues of RgIA. All peptides were synthesized by the standard Fmoc solid-phase synthesis strategy. To form the native CysI–CysIII and CysII–CysIV disulfide binding form (globular form), a two-step oxidation scheme was applied. The first pair of cysteine residues (CysI–CysIII) was linked by treatment with ferricyanide, and the second disulfide bond (CysII–CysIV) was then formed by iodine oxidation. Subsequently, all bicyclic peptides were purified by preparative reversed-phase high performance liquid chromatography (RP-HPLC). The synthesized products were identified using analytical RP-HPLC and electrospray ionization mass spectrometry (ESI-MS). The liquid chromatogram and mass spectrum of RgIA and peptide **13** are shown in Figure 2. The RgIA has a retention time of 13.64 min (min), whereas **13** is more hydrophobic, with a retention time of 14.36 min (Figure 2A,C). The observed average molecular mass (1571.13 Da) of RgIA and **13** was consistent with the theoretical mass (1590.79 Da) (Figure 2B,D). The additional analogues were synthesized by the same protocol.

### 2.2. Potency of RgIA and d-Amino Acid Substitutions at the Rat and Human α9α10 nAChRs

**Figure 3:**
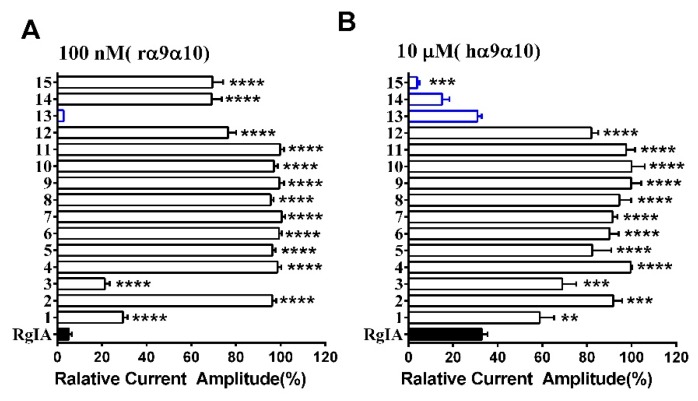
RgIA and its D-scan substitution were tested on rat and human α9α10nAChR. (**A**) ACh-evoked current inhibition of rat α9α10 nAChR by D-scan analogues (open bars) versus native RgIA (filled bar) at 100 nM; (**B**) ACh-evoked current inhibition of human α9α10 nAChR by RgIA (filled bar) and its analogues (open bars). Analogues incorporating higher or similar inhibition activity compared with wild-type peptide l (blue bars). The difference between the relative current amplitude of RgIA and each mutant was evaluated using one-way analysis of variance (ANOVA) followed by Dunnett’s t test; and the P values are indicated as follows: * *P* < 0.05; ** *P* < 0.01; *** *P* < 0.001; **** *P* < 0.0001. Error bars represent the mean ± SEM (*n* = 3–11).

The effect of l-to-d amino acid conversion in RgIA analogues was determined on the rat and human α9α10 nAChRs by using a method of two-electrode voltage clamp recordings. Firstly, all variants were tested on rat nAChR at 100 nM. Meanwhile, they were also investigated on human α9α10 at a concentration of 10 μM because previous research proved that RgIA had a lower inhibition on human nAChR [22]. For the rat α9α10 nAChR, most analogues caused little inhibition or none, except for **1**, **3**, and **13**. Strikingly, peptide **13** inhibited 97.2 ± 0.05% (*n* = 3) of current amplitude, even exceeding native RgIA, whereas analogues **1** and **3** had a lower activity, with 70.6 ± 2.0% (*n* = 5) and 78.7 ± 2.0% (*n* = 6) inhibition, respectively (Figure 3A). For human α9α10 nAChR, only three analogues, namely **13**, **14**, and **15**, retained similar activity relative to the wild-type RgIA, which produced about 70–90% inhibition of ACh-evoked currents at 10 μM (Figure 3B). More impressively, peptides **14** and **15**, of which l-Args were mutated to d-Args, preserved the inhibition affinity of human α9α10. On the contrary, they did not inhibit ACh-evoked currents of rat α9α10 nAChR (Figure 3).

**Figure 4:**
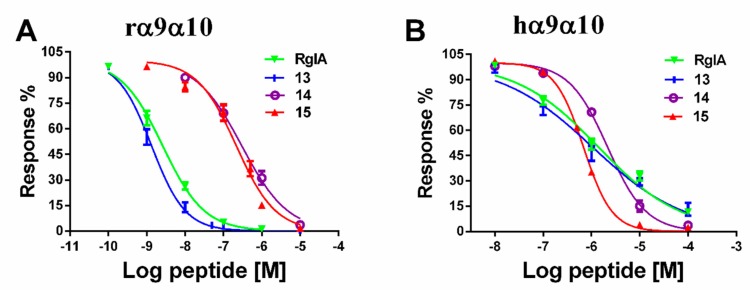
Concentration-response curves of RgIA and its D-amino acid substitutions for inhibition of α9α10 nAChR. (**A**) RgIA and its mutants were applied to rat α9α10 nAChR; (**B**) Peptides were tested on human α9α10 nAChR. Values are mean ± SEM from 5–11 separate oocytes.

The next step was to investigate the concentration-response relationships of analogues. The IC_50_ values of these mutants were determined on rat and human α9α10 subtypes (Table 1). Substitution of the l-amino acid residue with the corresponding d-enantiomer had a substantial impact on the inhibition activity of RgIA analogues. The IC_50_ values of most analogues displayed an obvious elimination or decrease in activity. Specifically, five analogs (**4**, **5**, **6**, **7**, and **11**) were not active at a concentration of 10 μM. For another five mutants (**2**, **8**, **9**, **10**, and **12**), affinity decreased for rat α9α10 nAChR more than 200-fold. In contrast, peptides **1** and **3** retained partial activity, with 41.07 nM and 31.77 nM of IC_50_, respectively. Strikingly, peptide **13** was the most potent mutant, with an IC_50_ of 1.33 nM, which was nearly two-fold compared with that of native RgIA (Figure 4). In addition, it is noteworthy that peptides **14** and **15**, in which all l-Args were substituted by d-Args, still had IC_50_ values of 215.7 nM and 288.5 nM, respectively (Figure 4).

We also evaluated the binding affinity of all analogues on human α9α10 nAChR. Among them, a similar tendency was observed, and a total of 10 out of 15 mutants were not active. Peptides **3** and **8** had a deduction in IC_50_ values, which were 6440 nM and 6950 nM, respectively. Peptide **13** maintained a similar potency to RgIA, with an IC_50_ value of 1050 nM, whereas peptide **14** exhibited an IC_50_ of 2170 nM. Mutant **15** displayed an increased potency on human α9α10, with an IC_50_ value of 680 nM. Concentration-response relationship curves of critical analogues (**13**, **14**, and **15**) are shown for comparison in Figure 4.

Furthermore, we also assessed the pharmacological selectivity of these mutants against other nAChR subtypes. **13**, **14**, and **15** were inactive against the rat α3β2, α4β4, α4β2, α2β4, α6/α3β4, and mouse α1β1δε nAChR subtypes according to the data from electrophysiological assays (data not shown).

### 2.3. Stability of RgIA and Its d-Amino Acids Substitutions

**Figure 5:**
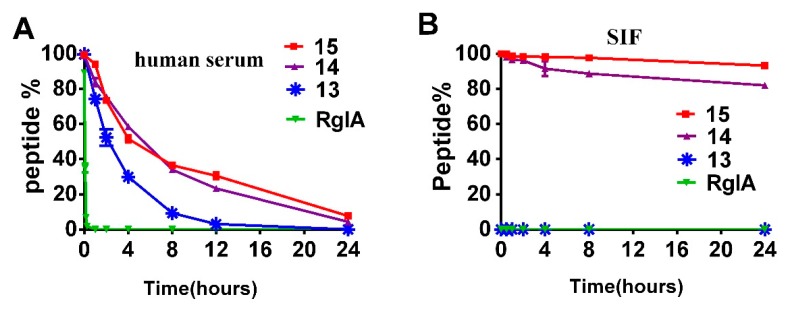
Relative stability of RgIA, **13**, **14**, and **15**. (**A**) Stability of RgIA, and peptides **13**, **14**, and **15** in human serum. (**B**) Stability of RgIA, **13**, **14**, and **15** in the SIF. Error bars represent the mean ± SEM (*n* = 3).

In the next series of experiments, Reverse-phase Ultra Performance Liquid Chromatography (RP-UPLC) was applied to investigate the degradation rate of RgIA, and peptides **13**, **14**, and **15** in human serum and simulated intestinal fluid (SIF). Firstly, we compared the stability of these peptides in human serum. The data showed that the half-life of RgIA was approximately 3 min and it was degraded completely in 15 min (Figure 5A). In contrast, peptide **13** containing D-Arg at the C-terminus was more stable, with the half-life of 120 min (Figure 5B). To our surprise, a significant enhancement of stability was observed for peptides **13** and **15**, with a half-life of approximately 4 h. We subsequently examined the stability of these peptides in SIF. The data showed that the native RgIA and **13** were decomposed immediately in SIF. On the contrary, even after 24 h, only 10%–20% of peptides **14** and **15** degraded in SIF. These results suggest that all l-Arg residues replaced by their d-enantiomers in RgIA markedly enhanced its stability.

### 2.4. Circular Dichroism (CD) Spectra of RgIA and Its Mutants

**Figure 6:**
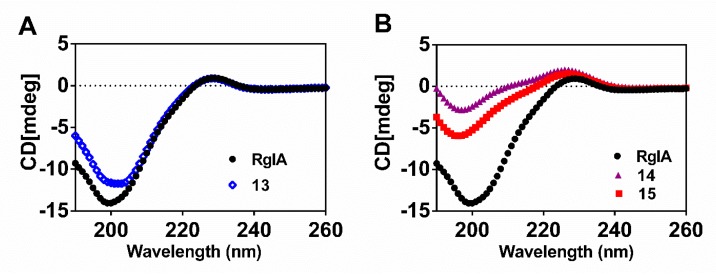
Representative CD spectra of wild-type RgIA and its variants. (**A**) The spectra of the native RgIA and peptide **13**; (**B**) The spectra of the native RgIA, and peptides **14** and **15**.

Previous research revealed that RgIA has a type I β-turn from Cys2 to Asp5 in Loop I. On the contrary, Loop II is less well-defined, where Tyr10-Cys12 forms an inverse γ- turn [30,31]. Here, the secondary structure conformation of critical d-scan analogues was compared with wild-type RgIA using CD spectra analysis (Figure 6). The results reveal that **13** (blue diamonds), for which the C-terminal l-Arg residue was replaced by the d-Arg residue, has a similar spectral curve to RgIA (black circles). This occurrence demonstrates that peptide **13** preserved the approximate secondary structure, which is consistent with the previous study that showed that Arg-13 truncation did not affect the activity of RgIA [30]. Correspondingly, the spectra of peptides **14** and **15** show little structural change comparable to that of native peptide, which indicates that they tend to produce a helical structure. These results were as expected because the peptides were mainly stabilized by two disulfide bonds and the little modification did not impact its integral structure remarkably. Similar results have been reported for some other conotoxins [32,33].

### 2.5. Molecular Modeling

**Figure 7:**
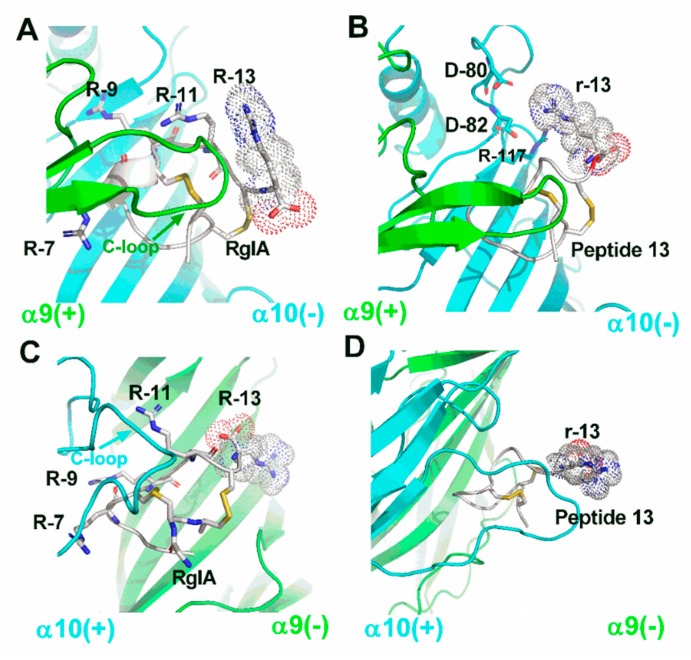
Molecular models of the interactions between wildtype RgIA, peptide **13**, and rat α9α10 nAChR. The α9 subunit is drawn in green, the α10 subunit is in cyan, and the peptides are in white. (**A**) RgIA bound with the rat α9(+)α10(–) interface, where all arginine residues in RgIA are labeled. (**B**) The molecular model is shown between peptide **13** and α9(+)α10(–) interface, and amino acids around 4 Å radius of the **r**-13 are labeled. (**C**) RgIA bound with rat α10(+)α9(–) interface, where all arginine residues in RgIA are labeled. (**D**) The molecular model is shown between peptide **13** and α10(+)α9(–) interface. All images were produced by PyMOL.

To understand the interaction between the peptides and rat α9α10 nAChR, four molecular dynamic (MD) simulation models of RgIA/α9(+)α10(−), peptide **13**/α9(+)α10(–), RgIA/α10(+)α9(−), and peptide **13**/α10(+)α9(−) complexes were constructed. Each model system was generated by a 50 ns molecular dynamics simulation. In the α9(+)α910(–) binding interface, R-13 of RgIA is on the outside of the complex with high mobility, and the interaction between R-13 and the receptor is weak. A similar result is also found in the peptide **13**/α9(+)α10(−) model (Figure 7A,B). Our model shows that D-40, D-42, and R-117 of rat α10 form charge−charge interactions, which may have led to the potency increase of peptide **13**; however, these interactions are short-lived as a result of the high mobility of **r**-13 (Figure 7B). In the α10(+)α9(−) binding interface, R-13 of RgIA and **r**-13 of peptide **13** have a similar orientation and still occur on the outside of models with high mobility (Figure 7C,D). These results may explain why RgIA still retained bioactivity when its Arg-13 was deleted. By contrast, our MD simulation results were consistent with a previous study concerning the molecular mechanisms between RgIA and the α10 (+) α9(−) interface [16]. In addition, all molecular modeling results indicated that three arginine residues (R-7, R-9, and R-11) buried in the binding site have critical electrostatic interactions with residues of the α9/α10 subunit. Meanwhile, the main contact between loop I of RgIA and the C-loop of the α9 or α10 subunit was also demonstrated in our models. Therefore, the chirality conversion of these residues probably altered the noncovalent interaction between RgIA and the α9/α10 subunit, which resulted in the loss of activity at α9α10 nAChR. However, our models cannot explain why peptides **14** and **15** still retain partial activity against the human and rat α9α10 nAChR. More intensive research should be done to clarify this.

## 3. Discussion

We synthesized a series of RgIA analogues containing one or more d-amino acids and evaluated their potency for inhibiting rat and human α9α10. Furthermore, we determined the selectivity of the more potent analogues. In addition, we performed serum and SIF digestion assays to screen their increased stability. These studies provided insights into the important side-chain chirality of amino-acid residues for biological function in RgIA. Moreover, several mutants retained full activity at the α9α10 nAChRs and exhibited improved serum and SIF stability over the native RgIA.

In the past decade, alanine-scanning and the site mutagenesis method have been widely used to provide insights into the role of the side-chain function of specific residues in many peptides [34]. Similarly, they have been adopted for several α-CTxs, including Vc1.1, AuIB, RegIIA, and TxID, to reveal the critical amino acids for the nAChRs blockade [35,36,37,38,39]. The systematic replacement of l-amino acid residues with their d-enantiomers provides another scheme to explore conformational effects in bioactive peptides. Several models of d-scans provided more knowledge on the effect of amino acids chiral conversion [19,34,40]. In this study, the substitution of residues in Loop I and loop II with d-amino acids led to a dramatic decrease or abolishment of biological activity in accordance with Ellison’s previous research. Ellison investigated the structure-activity relationship of RgIA using site mutagenesis, and the result revealed that four residues (Asp-5, Pro-6, and Arg-7 in the loop I, Arg-9 in the loop II) were crucial for its high-affinity binding to the α9α10 nAChR [30]. The reason for the loss of activity may be the conformational changes, which were induced by the conversion of l- to the d-amino acids. In addition, we substituted four cysteines, which are essential for forming disulfide bonds, with d-enantiomers that cannot be realized using the traditional alanine-scanning strategy. Electrophysiology results showed that these analogues still blocked rat α9α10 nAChR at a high concentration. Especially for peptide 3, it had robust inhibitory activity against the rat α9α10, with an IC50 of 31.77 nM. On the contrary, the other three analogues, **2**, **8**, and **12**, had a substantially decreased binding affinity. In the future, the structures of these four analogues need to be clarified, which will contribute to elucidating the molecular mechanism between them and rat α9α10 nAChR.

The electrophysiological data showed that most mutants displayed a dramatic loss of activity. However, the analogue **13** still exhibited a high potency, with an IC_50_ of 1.33 nM against rat α9α10 nAChR compared with the parent peptide. The Arg-13 is at C-terminal and out of the disulfide bond interlock region in RgIA. MD simulations showed that Arg-13 is located on the outside of models with high mobility. This phenomenon may explain why only peptide **13** preserved the full capacity for inhibiting rat α9α10 nAChR. These results suggested that d-amino acid substitution at the flank of the peptide would be a better choice for the design of peptides. Interestingly, a paradoxical phenomenon was found, as peptides **14** and **15** still blocked human α9α10 nAChR with a micromole IC_50_, while other mutants, even with one-Arg substitutions (compounds **1**, **3**, and **6**), completely lost activity towards the human α9α10 nAChR. As mentioned above, an increase in the helix content is observed in peptides **14** and **15**. We speculate that these d-amino acids may alter the spatial configuration of peptides and thus the selectivity of peptides are affected. Therefore, the molecular mechanism of ligands acting with receptors is more complicated than previously imagined. Further studies on three-dimensional structures of the mutants may help to explain the effect of d-amino acid substitutions in conotoxins. After clarifying the key stereocenters of RgIA, the selectivity of peptides **13**, **14**, and **15** was assessed against other nAChR subtypes. The result indicated that the three analogues still preserve their high selectivity, similar to RgIA.

The apparent drawback of RgIA as a drug is its poor stability and short biological half-life in vivo. Consequently, two studies tried to enhance its stability by chemical modification. Backbone cyclization was used to improve its stability by Halai et al. and it has been applied to many conotoxins [25]. Another study demonstrated that replacing disulfide bridges with a nonreducible dicarba bridge in RgIA can also increase its stability in serum [24]. However, the complicated synthetic steps and low yield put these methods at a disadvantage. As we know, natural peptides and proteins are almost homochiral polymers with l-amino acids exclusively. Substituting l-amino acids with d-isomers in the peptide will change its activity and improve stability against proteolytic attack. So far, several applications of this substitution approach have been made in other peptides. One study demonstrated that desmopressin containing a d-Arg was more stable with the mean terminal half-life of 3.7 h [41]. Another example is the MUC2 epitope peptide, where the d-amino acid-substituted analogues exhibited a high resistance in human serum and lysosomal preparation [28]. Till now, however, this strategy has not been applied successfully in the field of conotoxin research. In this study, the d-Arg substituted peptide **13** showed an increased serum stability, but it degraded fast in SIF, similar to RgIA. More impressively, peptides **14** and **15** were highly stable in human serum and SIF; especially for peptide **15**, which displayed a two-fold increase in the inhibition of humanα9α10 nAChR compared to RgIA. Therefore, the d-amino acid-substituted chemistry strategy is an option for redesigning these peptides to benefit the future development of conopeptide-based therapeutics.

## 4. Experimental Procedure

### 4.1. Peptide Synthesis 

All peptides were synthesized using standard Fmoc (N-(9-fluorenyl)methoxycarbonyl) chemistry as described previously [42]. The RgIA and analogues **1**–**12** were prepared on Fmoc-l-Arg(Pbf)-Wang resin, while peptides **13** and **14** were carried out on Fmoc-d-Arg(Pbf)-Wang resin. The peptide **15** was synthesized on Rink amide resin. For peptides **1**–**13**, each residue of RgIA was systematically replaced by its corresponding D-amino acid, and all arginine residues in peptides **14** and **15** were substituted with d-Args. In order to form two correct disulfides (CysI- CysIII, CysII-CysIV), we used a combination of trityl(Trt) and acetamidomethyl(Acm) for side chain protection of cysteines. Briefly, the linear peptides were deprotected from the resin by adding Reagent K (trifluoroacetic acid/water/ethanedithiol/phenol/thioanisole; 90:5:2.5:7.5:5, v/v/v/v/v) for 2 h at room temperature. Then, the resin was removed by filtration and the released peptides were precipitated and washed with cold ether. After lyophilization, the crude peptides were purified using a Vydac C18 column (300 Å, 5 µm, 10 mm × 250 mm) on a Wasters 2535 HPLC. Afterwards, the linear peptides were folded to form a disulfide using a two-step oxidation strategy. The first disulfide was formed under an oxidative condition using 20 mM potassium ferricyanide and 0.1 M Tris-HCl, pH 7.5, which reacted for 45 min, and the monocyclic peptide was purified by RP-HPLC. Then, the Acm groups were cleaved and form the second disulfide by iodine oxidation, as previously described [42]. All bicyclic products were purified by RP-HPLC. The purity of synthesized bicyclic peptides was determined by analytical RP-HPLC and monitored by absorbance at 214 nm (≥ 95% purity). Meanwhile, ESI-MS was utilized to confirm the identity of the products.

### 4.2. In Vitro cRNA Synthesis

The cDNAs of rat and human nAChR subunits used in this study were kindly provided by Stefan H. Heinemann (Salk Institute, San Diego, CA, USA). The plasmids of human and rat nAChR subunits were linearized with an appropriated restriction enzyme ((TaKaRa, Japan)). Capped RNA for these subunits was synthesized with the T7 mMessage mMachine transcription kit (Ambion, Austin, TX, USA) in vitro, and then purified using the MEGA clear^TM^ Transcription clean-up Kit ((Invitrogen; Thermo Fisher Scientific, Inc., Austin, TX, USA). The concentration of each cRNA was determined at 260 nm by a Smart SpecTM plus Spectrophotometer (Bio-Rad, USA). cRNAs of the various subunits were combined to produce 200−500 ng/μL for each subunit of cRNA. Then, 50-nL aliquots were injected into each *Xenopus* oocyte with a Drummond microdispenser (Drummond Scientific, Broomall, PA, USA), and incubated at 18 °C in ND_96_ buffer (96 mM NaCl, 2 mM KCl, 1.8 mM CaCl_2_, 1 mM MgCl_2_, and 5 mM HEPES, pH 7.4) including 10 mg/L penicillin, 10 mg/L streptomycin, and 100 mg/L gentamicin.

### 4.3. Electrophysiology Measurements

Two electrode voltage clamp recordings from oocytes were performed using an Axoclamp 900A amplifier (Molecular Devices Corp., Sunnyvale, CA, USA) and Clampfit 10.2 software (Molecular Devices Corp., Sunnyvale, CA, USA). In the first place, oocytes were placed in the 50 μL recording chamber and then perfused with the ND96 solution containing 1 μM atropine and 0.1 mg/mL bovine serum albumin (BSA). For the α9α10 and mouse muscle α1β1δε subtypes, the ND-96 solution did not contain atropine. The oocytes were clamped with a holding potential of −70 mV at room temperature. Oocytes were voltage-clamped and exposed to acetylcholine (Ach) and peptide variants as described previously [43]. All peak current amplitudes data were acquired using an Axon Digidata 1550 Data Acquisition System (Molecular Devices Corp.). Data were sampled at 500 Hz and filtered at 10 Hz.

### 4.4. Date Analysis

The one-way ANOVA/Dunnett’s post-test were used for statistical analysis with GraphPad Prism 6.0 software (GraphPad Software, San Diego, CA, USA). Error bars are given as mean ± SEM. Asterisks represent a significant difference between RgIA and its mutants. (* *P* < 0.05, ** *P* < 0.01, *** *P* < 0.001, and **** *P* < 0.001). The concentration-response data were fitted to the equation, % response = 100/(1 + ((toxin)/IC50)nH), using GraphPad Prism. Each data point of a concentration-response curve represents the average ± SEM of measurement from at least 5–11 oocytes, where nH is the Hill coefficient. All IC_50_ values were calculated by GraphPad Prism (GraphPad Prism Software Inc., San Diego, CA, USA).

### 4.5. Stability Assays

Stability assays of RgIA and analogues **13**, **14**, and **15** were carried out on male AB human serum (Sigma-Aldrich, Germany) and simulated intestinal fluid (SIF). Firstly, the serum was centrifuged at 15,000 rpm for 15 min to remove lipids and it was incubated for a further 15 min at 37°C before the assay. Triplicate peptide samples were dissolved in serum at a concentration of 100 μM and incubated at 37 °C. The 40 μL aliquots of analogues **13**, **14**, and **15** were taken out at 0, 0.5, 1, 2, 4, 8, and 24 h, whereas RgIA was taken out at 0, 5, 10, and 15 min. Each aliquot was denatured with 40 μL of 6 M urea and incubated for 10 min at 4°C. Then, 40 μL of 20% trichloroacetic acid (TCA) was added to precipitate serum proteins for another 10 min at 4°C. The precipitated serum proteins were removed using centrifuging at 14,000 rpm for 10 min, and 10 μL of the supernatant was analyzed on ultra-performance liquid chromatography (RP-UPLC). SIF was prepared as described in the U.S. Pharmacopeia. RgIA and its analogues were added to SIF at a concentration of 100 μM (in triplicate) and incubated at 37 °C. Following this, 50 μL aliquots were taken at 0, 1, 2, 4, 8, 12, and 24 h, quenched with 50 μL of 4% aqueous trifluoroacetic acid, and analyzed by RP-UPLC. The amount of remaining peptides was quantified by measuring the peak area.

### 4.6. Circular Dichroism (CD) Spectroscopy

CD spectra were recorded on a Chirascan CD spectrometer (Applied Photophysics, Leatherhead, U.K.) at room temperature under constant nitrogen flush. The peptide solutions were dissolved in water at a concentration of 200 μM. The measurements were performed in a 1 mm quartz cuvette and the spectral data of peptides were recorded from far-UV (190 to 260 nm) with a step size and a bandwidth of 1 nm. The spectra were obtained from the average of five measurements after subtraction of the background signal. The spectra were expressed as units of molar ellipticity ([θ]) using Applied Photophysics Chirascan software.

### 4.7. Molecular Modeling

Molecular models of the interaction between RgIA/**13** and rat α9(+)α10(–)/α10(+)α9(–) nAChRs were created using Modeller9v13 [44]. Briefly, the structure of d-Arg was constructed using the ChemBioDraw program. The initial structure of **13** was built by replacing the l-Arg-13 residue with constructed d-Arg at the corresponding position using GaussianView and the UCSF Chimera 1.12 package. Then, the spatial structures of the isolated human α9 subunit (PDB code: 4UY2) and α4β2 nAChR (PDB code:5KXI) were chosen as templates to generate a detailed conformation of the peptide-binding rat α9α10 nAChR subunit [45,46]. The homology models were then refined using the Gromacs 5.14 MD engine with the AMBER ff99SB force field [47,48]. The complexes were solvated in a cubic SPC/E water box of size 0.5 × 0.5 × 0.5 nM. Sodium ions were added to neutralize the electroneutral system. Before conducting the MD stimulations, the system was further minimized for 10,000 steps using the steepest descent algorithm for 1,000 ps. A second minimization was carried out, but with all position restraints withdrawn. The systems were then gradually heated up from 50 to 298.15 K in the NVT ensemble over 100 ps to constrain the position of atoms in the box. The 50 ns production runs were performed in an isobaric-isothermal (NPT) ensemble under a 298.15 K temperature and 1 bar pressure. Simulations were performed with the AMBER ff99SB force field and prepared and performed with GROMACS 5.14. The MD trajectories were analyzed using the VMD program (http://www.ks.uiuc.edu/). Then, the figures were drawn using PyMOL (http://www.pymol.org).

## 5. Conclusions

In summary, we replaced each l-amino acid of RgIA with the corresponding d-isomer systematically, and the two analogues **14** and **15** were also redesigned. The results indicated that this modification had a significant impact on the potency of inhibition towards α9α10 nAChR. Electrophysiological analysis and stability studies indicated that peptide **13** preserved high activity and selectivity for human α9α10 nAChR, as well as stability against enzymatic degradation. Remarkably, peptides **14** and **15** retained affinity for human α9α10 nAChR and showed unparalleled stability compared to native RgIA. An alternative vision was offered by this paper to understand the structure-function relationships between RgIA and α9α10 nAChR. Our approach provides assistance for the design and development of conotoxin analogues for stability improvement. Further studies are needed to identify the three-dimensional structures and illuminate the molecular mechanisms of analogues **13**–**15**. In addition, analgesic experiments in animal models should be conducted to evaluate these compounds in the near future.

## Figures and Tables

**Table 1:** Potencies of RgIA and its mutants on rat and human α9α10 nAChR subtypes expressed in *Xenopus laevis* oocytes.

Compound	α9α10 (Rat)		α9α10 (Human)	
	IC_50_ * (nM)	Hillslope	IC_50_ (nM)	Hillslope
RgIA	2.60 (2.10–3.22)	0.80 (0.68–0.92)	1400 (1000–1960)	0.51 (0.42–0.60)
**1**	41.07 (31.67–53.27)	0.78 (0.65–0.92)	>10000	-
**2**	705.4 (478.8–1039)	0.56 (0.42–0.70)	>10000	-
**3**	31.77 (26.66–37.87)	0.84 (0.71–0.97)	6440 (2790–1490)	0.51 (0.24–0.77)
**4**	>10000	-	>10000	-
**5**	>10000	-	>10000	-
**6**	>10000	-	>10000	-
**7**	>10000	-	>10000	-
**8**	2129(1697–2671)	0.93(0.76–1.11)	6950 (4500–10700)	1.32 (0.51–2.13)
**9**	1752 (1351–2272)	1.02 (0.77–1.26)	>10000	-
**10**	6012 (4747–7614)	0.88 (0.69–1.08)	>10000	-
**11**	>10000	-	>10000	-
**12**	533.4 (389.9–729.6)	0.71 (0.56–0.85)	>10000	-
**13**	1.33 (1.086–1.638)	0.99 (0.80–1.17)	1050 (700–1590)	0.46 (0.37–0.54)
**14**	215.7 (170–273.8)	0.85 (0.68–1.02)	2170 (1800–2580)	1.09 (0.92–1.27)
**15**	288.5 (224.9–398.9)	0.72 (0.59–0.86)	680 (650–720)	1.47 (1.32–1.62)

* Numbers in parentheses are 95% confidence intervals, and all data calculated by GraphPad Prism from the values of 3−11 oocytes (*n* = 3–11).
